# Activation of Auditory Cortex by Anticipating and Hearing Emotional Sounds: An MEG Study

**DOI:** 10.1371/journal.pone.0080284

**Published:** 2013-11-20

**Authors:** Koichi Yokosawa, Siina Pamilo, Lotta Hirvenkari, Riitta Hari, Elina Pihko

**Affiliations:** 1 Brain Research Unit, O.V. Lounasmaa Laboratory, and MEG Core, Aalto NeuroImaging, School of Science, Aalto University, Espoo, Finland; 2 Faculty of Health Sciences, Hokkaido University, Sapporo, Hokkaido, Japan; Baycrest Hospital, Canada

## Abstract

To study how auditory cortical processing is affected by anticipating and hearing of long emotional sounds, we recorded auditory evoked magnetic fields with a whole-scalp MEG device from 15 healthy adults who were listening to emotional or neutral sounds. Pleasant, unpleasant, or neutral sounds, each lasting for 6 s, were played in a random order, preceded by 100-ms cue tones (0.5, 1, or 2 kHz) 2 s before the onset of the sound. The cue tones, indicating the valence of the upcoming emotional sounds, evoked typical transient N100m responses in the auditory cortex. During the rest of the anticipation period (until the beginning of the emotional sound), auditory cortices of both hemispheres generated slow shifts of the same polarity as N100m. During anticipation, the relative strengths of the auditory-cortex signals depended on the upcoming sound: towards the end of the anticipation period the activity became stronger when the subject was anticipating emotional rather than neutral sounds. During the actual emotional and neutral sounds, sustained fields were predominant in the left hemisphere for all sounds. The measured DC MEG signals during both anticipation and hearing of emotional sounds implied that following the cue that indicates the valence of the upcoming sound, the auditory-cortex activity is modulated by the upcoming sound category during the anticipation period.

## Introduction

Humans detect positive and negative emotions easily from both linguistic and nonlinguistic utterances [1] as well as from environmental sounds, such as crashes, breaking of glass, and music. Emotional sounds are important for social interaction and bonding, but they also serve a survival value in reorienting the processing resources. In the visual modality, emotional pictures, compared with neutral pictures, can enhance the processing already in the early visual cortices [2]. The auditory cortices are also affected by emotion. For human voice, cortices associated with auditory function—in addition to several cortical and subcortical areas commonly related to emotional processes—react more strongly to emotional than neutral prosody [3]–[5]. The auditory-cortex responses to emotional sounds may appear within 0.3 s from the beginning of the stimulus [6], [7], indicating the readiness for fast emotion detection. Some electrophysiological studies have shown subsequent slow shifts up to 0.5 s after the onset of an emotional sound [8], [9]. In addition to human voice, other types of complex emotional sounds lead to increased activation of the auditory cortices [10]. Even neutral tones conditioned in advance to emotional valence affect the auditory-cortex 100-ms neuromagnetic response N100m [11].

Anticipation of an imperative stimulus, cued by a preceding stimulus, can evoke slow scalp-negative EEG potentials [12]–[16] that are also sensitive to the anticipation of emotional pictures [17]–[19]. This slow shift consists of an earlier, orienting part occurring soon after the warning stimulus and of a later response reflecting, depending on the task, motor preparation or, when no motor action is required, anticipatory attention or cognitive preparation to the second stimulus [13], [14], [20]–[22]. Studies using magnetoencephalography (MEG) and source analysis suggest that during this later, anticipatory phase, the sensory cortex to be stimulated is already active. Thus, for example, during anticipation of an auditory imperative stimulus cued by a visual stimulus, the auditory cortex can be activated already during the later anticipation period, within 0.5 s before the auditory stimulus sound [23].

The aim of the current study was to determine whether anticipation of emotional vs. neutral sounds would modulate the activation of auditory cortices similarly during the early and late parts of the anticipation period and during listening to the sounds. We used MEG to obtain excellent temporal and good spatial resolution in the study of auditory-cortex activation, and we measured auditory evoked magnetic fields without applying high-pass filtering (direct current, DC) to reliably obtain both fast and slow brain signals. The 10.5-s time sequence included, after a 0.5-s baseline, a short cue tone followed after 2 s by a 6-s long emotion-evoking or neutral natural sound. The category of the upcoming sound (pleasant, neutral, or unpleasant) was indicated by the cue tone.

## Materials and Methods

### Subjects

Eighteen healthy volunteers participated in the experiment. Data from 3 subjects were excluded from the analysis: data from two subjects because of excessive eye blinking and data from one subject because of a questionable N100m source location. The final analysis was therefore based on data from 15 subjects (8 females, 7 males; mean ± SD age, 27.5±6.9 yrs; age range, 21–47 yrs; all right-handed).

### Ethics Statement

The MEG recordings were approved by the Ethics Committee of the Hospital District of Helsinki and Uusimaa, and written informed consent was obtained from each participant prior to the experiments.

### Stimuli

**Figure 1:**
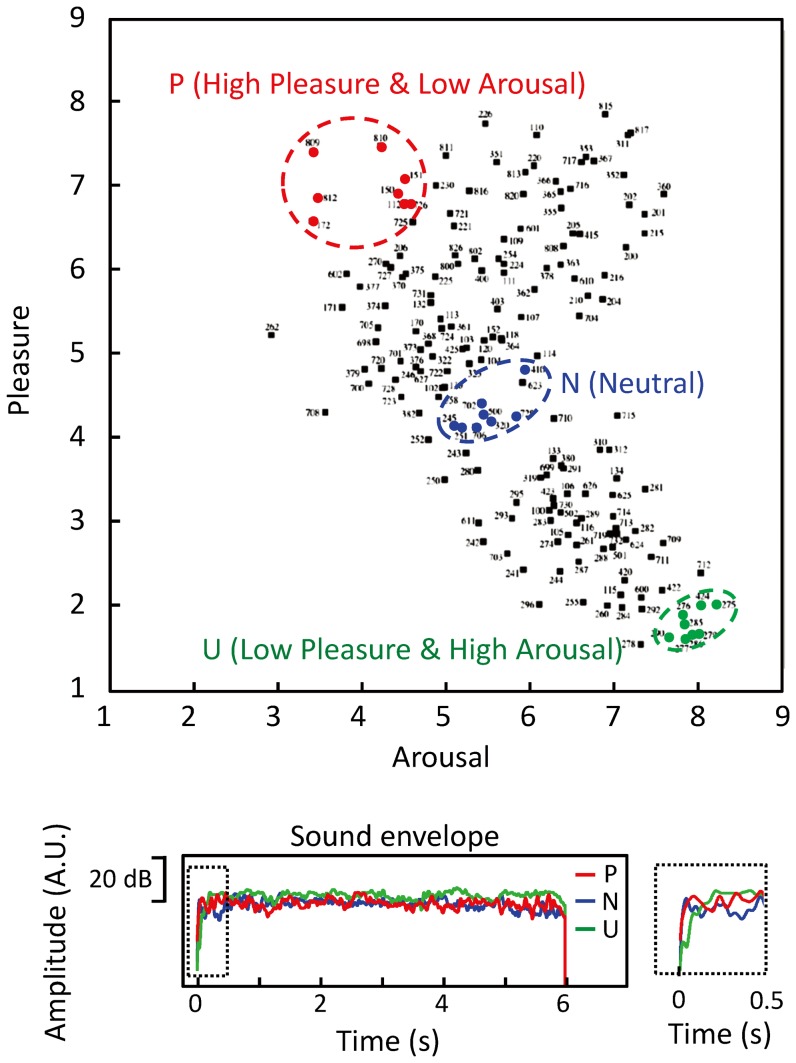
Profile of auditory stimuli. (top) Emotional sounds used as stimuli, shown in the valence and arousal matrix of IADS-2. Sounds used for high pleasant and low arousal (P) category, neutral (N) category, and low pleasant and high arousal (U) category are marked with red, blue, and green dotted lines, respectively. (bottom) Amplitude envelopes averaged over 8 sounds belonging to the same category; whole duration (lower left) and details of the onset (lower right).

From the International Affective Digitized Sounds database (2nd Edition; IADS-2, University of Florida), we selected sounds that have been validated for emotional content by more than 100 listeners: eight “pleasant & low arousal” (abbreviated as P; e.g., music, birdsong, etc.), eight “neutral” (N; e.g., typewriter, wind, etc.), and eight “unpleasant & high arousal” (U; e.g., scream, car crash, etc.) sounds. Fig. 1 (top) shows the selected sounds along the Pleasure–Arousal scales among the sounds of the database, and Table 1 specifies our stimuli in more detail.

**Table 1:** Contents and affective ratings of auditory stimuli adopted from IADS-2.

Category	Description	Sound No.	Pleasure	Arousal	Substance^*^
	Kids1	112	6.84	4.46	Chatter of happy children
**High-pleasure**	Seagull	150	6.95	4.38	Seagull's song
**Low-arousal**	Robin	151	7.12	4.47	Robin's song
	Brook	172	6.62	3.36	Babble of stream
Average	Cork pour	726	6.82	4.51	Cork pulled and liquid poured
Pleasure: 7.03	Harp	809	7.44	3.36	Harp playing
Arousal: 4.02	Beethoven	810	7.51	4.18	Classic music
	Choir	812	6.9	3.43	Choir's singing
	Hiccup	245	4.18	5.05	Hiccupping
**Neutral**	Nose blow	251	4.16	5.14	Blowing one's nose
	Office1	320	4.23	5.48	Typing and phone's ring
	Helicopter2	410	4.86	5.89	Propeller's sound
Average	Wind	500	4.32	5.4	Wind blowing
Pleasure: 4.33	Belch	702	4.45	5.37	Burping
Arousal: 5.43	War	706	4.16	5.3	Machineguns and battle plane
	Paper2	729	4.3	5.79	Tearing paper
	Scream	275	2.05	8.16	Screaming of a woman
**Low-pleasure**	Fem scream2	276	1.93	7.77	Screaming of another woman
**High-arousal**	Attack1	279	1.68	7.95	Screaming and beating
	Attack2	285	1.8	7.79	Screaming and beating
Average	Victim	286	1.68	7.88	Screaming and gun shot
Pleasure: 1.88	Fight1	290	1.65	7.61	Arguing voice
Arousal: 7.83	Tire skids	422	2.22	7.52	Brake-screeching
	Car wreck	424	2.04	7.99	Brake-screeching and car crash

* Added by the authors.

Each sound was adjusted (cut at the end, when needed) to last 6 s and modified so that no sound had rise and fall times shorter than 10 ms. Additionally, the stimuli were normalized so that their maximum sound pressures were the same. Fig. 1 (bottom) shows the averaged sound envelopes for the three sound categories, indicating a slightly slower rise for U than for N and P sounds within the first 0.2 s but very similar sound intensities after 0.5 s.

A 100-ms cue tone with rise and fall times of 10 ms was presented 2 s before each emotional or neutral (P/N/U) sound. The pitch of the cue was 500 Hz, 1 kHz, or 2 kHz, indicating different valences of the upcoming sounds; the connection between the cue and the emotional sound was fixed for each subject but was counterbalanced across subjects. That is, the original 18 subjects were allocated evenly across the 6 different cue–stimulus combinations. The onsets of the successive cue sounds were separated by 20 s. Before the main experiment, the subjects participated in a 6-min training session to learn the relationship between the cue tones and the valence of the upcoming emotional sound category.

Consequently, each epoch consisted of an anticipation period (0–2 s; cue at time 0) and a hearing period (2–8 s). Both the cues and emotional sounds were presented via a non-magnetic speaker located in front of the subject in a magnetically shielded room.

All subjects were studied in two approximately 20-min sessions, each containing 60 cue–stimulus epochs—with the P, N and U sounds presented in a random order—and a few oddball epochs. The oddball epochs included a 40-ms burst of white noise at an arbitrary location of the cue–stimulus epoch. The subject's task was to count the number of oddball epochs in each session; these epochs were excluded from the analysis. This task was added to help the subjects to attend to the sounds and to keep their vigilance stable. Responses were thus collected for altogether 2×60 = 120 epochs, resulting in 40 epochs per sound category.

### Recordings

MEG signals were recorded with a 306-channel whole-scalp neuromagnetometer (VectorView™, Elekta Neuromag Oy, Helsinki, Finland) at the MEG Core of Aalto NeuroImaging, Aalto University, Espoo, Finland. The passband was from DC to 200 Hz, and the signals were sampled at 600 Hz.

### Analysis

The MEG signals obtained in the two sessions were merged off-line after conversion of the data into the same reference head position. Event-related signals from the 204 gradiometers (two orthogonal sensors at each of the 102 locations in the sensor helmet) were then averaged separately for each stimulus category, excluding the oddball epochs.

Because of the tonotopic organization of the auditory cortex, the source location of the 100-ms response N100m varies slightly according to the stimulus frequency [24]. However, to obtain a higher signal-to-noise ratio and a robust source location, we calculated the N100m source location for the cue tones by using the MEG signals averaged over the three cue tones (500 Hz, 1 kHz, and 2 kHz). Two equivalent current dipoles (ECDs), one in the left hemisphere and the other in the right hemisphere, were assumed in each individual brain. The locations and directions of the two equivalent current dipoles were calculated by “Source Modelling” software (Elekta, Neuromag) by using 20 pairs of orthogonal gradiometers over the temporal areas, i.e., 10 pairs for each hemisphere around the N100m maximum. The dipoles were fitted every 4.9 ms and the ECD with the highest goodness-of-fit values was selected. These sources were then used to explain the signals during the whole analysis period. The signal passband was 0.1–40 Hz for the analysis of the transient responses and DC–8 Hz for the slow shifts. Activations of the auditory cortical areas associated with anticipating or hearing emotional/neutral sounds were investigated by quantifying the slow shifts preceding the sounds as well as the sustained fields during the sounds. The mean N100m source location across the three cue tones (500 Hz, 1 kHz, and 2 kHz) was adopted as the source area for all signals because it is known that the auditory sustained field originates within 1 cm from the source of N100m (for example, [25]).

For each participant, the mean source strengths were computed within time windows of 0.2–0.35 s, 0.4–0.7 s, 1.0–1.5 s, and 1.5–2.0 s with respect to a baseline from −0.5 to 0 s before the cue onset and in time windows of 2.5–8 s with respect to a baseline from −0.2 to 0 s before the emotional-sound onset (i.e., from 1.8 to 2 s after cue onset).

Statistical significance of the source strengths was evaluated by testing the values against zero with one-way ANOVA followed by Tukey's multiple-comparison tests. Possible effects of cue tones as well as differences between hemispheres, time windows and emotional categories were analyzed with repeated measures of ANOVA (IBM SPSS Statistics 20). Greenhouse-Geisser correction was used when the sphericity was violated. The level of statistical significance was p<0.05.

## Results

### Source locations

**Figure 2:**
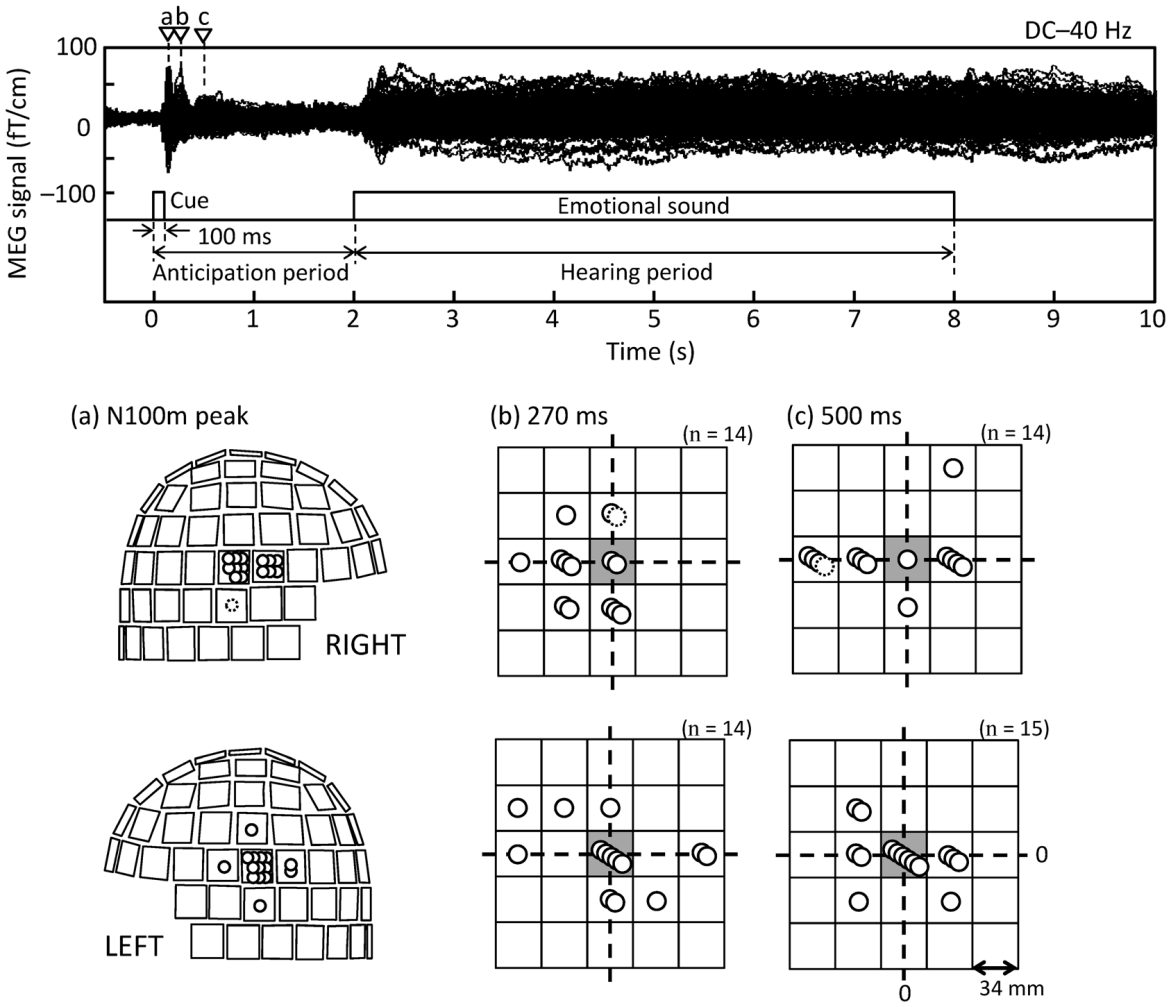
Time traces and maximum locations of magnetic field gradients. (top) An example of magnetic field gradients obtained by all gradiometers from one typical participant. Reverse triangles indicate the time points at the N100m peak (a) and broad peaks of 0.27 s (b) and 0.5 s (c). (bottom) (a) Positions of the gradiometer pairs that detected the strongest N100m signal in each individual; the helmet is shown from right (top) and left (bottom). (b, c) Sensor positions for the strongest 0.27-s (b) and 0.5-s (c) peaks given in spatial relationship to the sensor that picked the strongest N100m response in each individual (grey shading); the squares illustrate the sensor pairs (as in the helmets on the left), separated by 34 mm. Each stacked circle corresponds to one participant. Dotted circles refer to local maxima within the temporal area in a few cases with the global maximum located outside the temporal area. The signals were time-averaged over intervals from 0.2 to 0.35 s (b) and from 0.4 to 0.7 s (c), respectively. A few positions outside the temporal areas were eliminated from the figure.

Figure 2 (top) shows a “butterfly” display of the typical MEG waveforms of Subject 1. Transient deflections follow the onset of the cue and the onset of the emotional sound, and a sustained field with stable amplitude continues throughout the sound and even a few seconds afterwards.

The strongest transient responses were the N100m deflections, occurring bilaterally in sensors over the auditory cortices (Fig. 2a) and peaking on average 108 ms after the sound onset. The later responses, peaking around 0.27 s and 0.5 s, occurred in the vicinity of the strongest N100m (Figs. 2b and c). Therefore, the sources of N100m were used to explain also these later responses.

**Figure 3:**
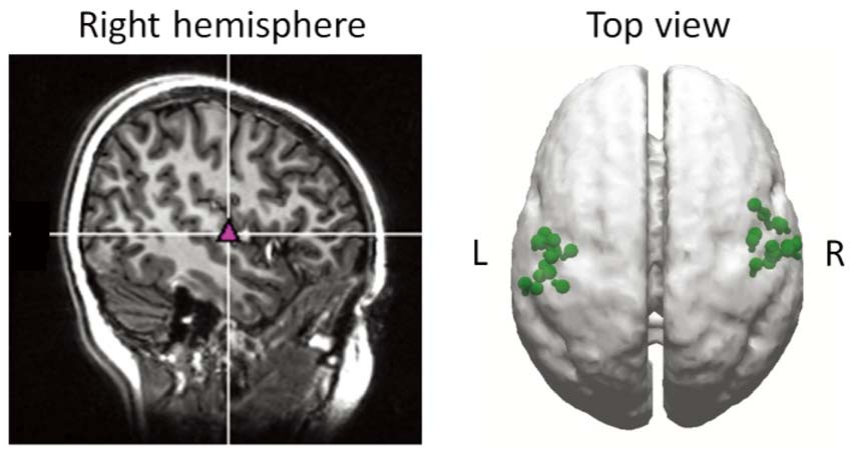
Locations of N100m sources to cue sounds. (a) Typical example of N100m source location superimposed on the anatomical image of one subject. (b) Distribution of the source locations of all participants superimposed on a top view of a template brain.

Figure 3 (left) shows the location of the current dipole for N100m to cue tone in the right supratemporal auditory cortex of Subject 2. This location agrees with many earlier reports (for reviews, [26], [27]). The N100m sources of all subjects clustered to the auditory cortices, as shown in Fig. 3 (right). The mean ± SD goodness-of-fit value of the dipole model was 94.5±4.6% (median, 95.8%), and all goodness-of-fit values exceeded 85%.

### Transient and sustained signals

**Figure 4:**
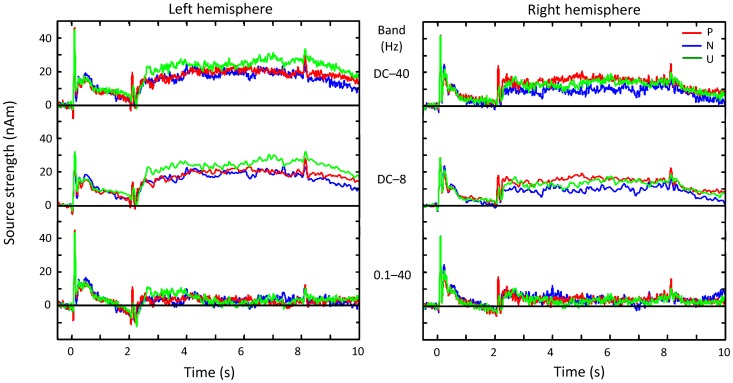
Time courses of grand-average source strengths across 15 subjects. Source strengths (i.e., dipole moments) are shown for full-band (0–40 Hz; upper row), low-pass-filtered (at 8 Hz; middle row) and high-pass-filtered (0.1–40 Hz; lower row) responses as a function of time from −0.5 to 10 s. The dipoles were assumed to have the same position and direction as sources of N100m. Baselines were set as the mean level between −0.5 and 0 s.

Figure 4 shows the grand-mean source waveforms of the MEG signals associated with the three sound categories (P/N/U) for the whole analysis period, separately for each hemisphere. The top traces show the source waveforms with passband from DC to 40 Hz, with prominent transient responses to the onsets of both sounds, decaying slow shifts (with peaks around 0.27 and 0.5 s) during the anticipation period, and prominent sustained fields throughout the emotional sound. Low-pass filtering at 8 Hz (middle traces) dampens the onset transients, and high-pass filtering at 0.1 Hz (bottom panels) strongly dampens the sustained fields. The DC–8 Hz traces were used for quantification of the slow shifts, and the 0.1–40 Hz traces were used for quantification of the N100m responses.

**Figure 5:**
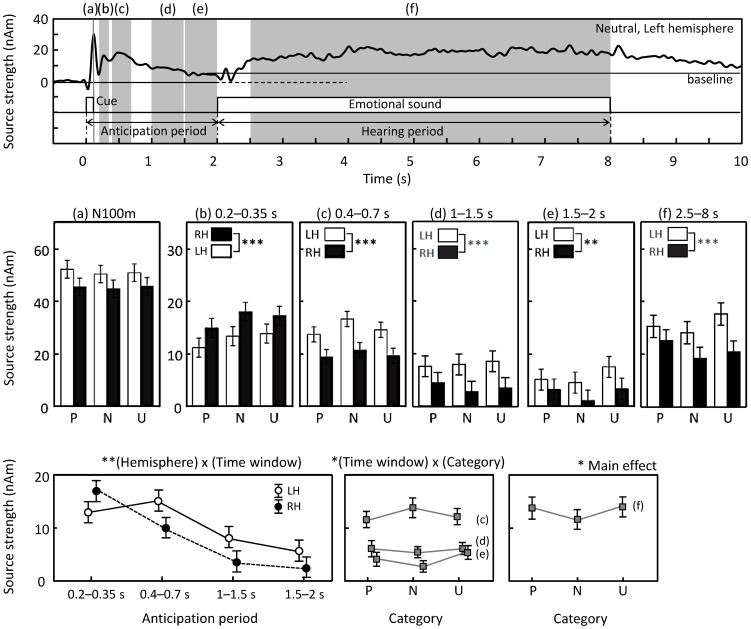
Results of statistical analysis. (top) Low-pass-filtered (at 8 Hz) response of the left hemisphere to neutral sounds (a trace from the middle left panel of Fig. 4). Shadowed belts (a)–(f) indicate time windows used for statistical analysis. (middle) Source strengths separately for the right (RH) and left (LH) hemispheres and sound categories within each time window (a)–(e). Baselines was from −0.5 to 0 s for the analysis of responses during the anticipation period (a)–(e), while it was from 1.8 to 2 s for the analysis of responses occurring during the hearing period (f). Mean ± 95% confidence intervals are shown across all 15 participants. (bottom) Left panel shows the interaction effect for *hemisphere* and *time window*, centre panel shows the interaction effect for *time window* and *category* in late anticipation period (c)–(e), and right panel shows the difference between categories during actual hearing (f). Mean ± SE (standard error) values are shown. * denotes *p*<0.05, ** *p*<0.01, *** *p*<0.001 in the figures.

Figure 5 shows a summary of the source strengths during the whole analysis period. The trace in the top panel is an example of left-hemisphere source waveform for neutral sounds, here used to illustrate the different analysis periods (shadowed belts a–f). In all time windows, the strengths of all sources in both hemispheres differed statistically significantly from zero.

### Anticipation period 0–2 s

First, the source strengths (dipole moments) and latencies of the N100m responses were analyzed. Main effects of *hemisphere* were observed both for source strength and latency (*hemisphere* (2) and *category* (3), *n* = 13; data of two participants not included because N100m responses were not clearly single-peaked) such that the dipole moments were stronger (mean difference, 13%; F(1,24) = 19.0, *p*<0.001) and the latencies were longer (mean difference, 1.9 ms; F(1,24) = 4.4, *p* = 0.047) in the left hemisphere (LH) than the right hemisphere (RH). For N100m, no main effects of *category* and interaction between *hemisphere* and *category* were observed.

After the N100m peak, subsequent source strengths gradually decreased during the anticipation period (Figure 5, main effect for *time window* (4), F(2,22) = 20.5, *p*<0.001 post-hoc linearity test *p*<0.001). The hemispheric laterality changed during the analysis period such that the activity was stronger in the RH during 0.2–0.35 s and in the LH during the rest of the time windows (middle row and left panel of bottom row in Figure 5; interaction between *hemisphere* (2) and *time window*, (4) F(2,25) = 7.5, *p* = 0.004, *post hoc* contrasts for hemisphere differences: 0.2–0.35 s vs. 0.4–0.7 s, *p* = 0.001; 0.2–0.35 s vs. 1–1.5 s, *p* = 0.006; 0.2–0.35 s vs. 1.5–2 s, *p* = 0.021).

To rule out the possibility of effects of cue tones on the period following N100m, we ran ANOVA with *cue tone* (3) and *hemisphere* (2) and *time window* (4), although the cue tones had already been counterbalanced across subjects. In addition to the main effect of *time window* (as with *category* as a factor, see above), an interaction was present for *time window* and *cue tone* (F(6,84) = 3.2, *p* = 0.007), suggesting that cue tones indeed had an effect on signal amplitudes, though not equal in all time windows. Separate ANOVAs indicated that *cue tone* had a main effect (F(2,28) = 5.6, *p* = 0.009) during the earliest time window (0.2–0.35 s) but no significant effect during the remaining time windows of the anticipation period (0.4–0.7 s, *p* = 0.06; 1–1.5 s, *p* = 0.8; 1.5–2 s, *p* = 0.4). Thus, to avoid interpretation problems, we eliminated the first time window (0.2–0.35 s) from further analysis. It should be noted that, despite a tendency of a cue effect in the 0.4–0.7-s time window (0.5 kHz>1 kHz>2 kHz), the direction of this tendency remained the same in 0.4–0.7-s and 1.5–2-s time windows, thus indicating no change towards the end of the anticipation period.

Analysis of just the three last time windows of the anticipation period (*hemisphere* (2) and *time window* (3) and *category* (3)) revealed, in addition to the main effects of *hemisphere* [F(1,14) = 10.5, *p* = 0.006] and *time window* [F(1.2,16.5) = 33.9, *p*<0.001], a significant interaction effect for *time window* and *category* [F(4,56) = 3.1, *p* = 0.022]. *Post hoc* comparisons contrasted the early time window (0.4–0.7 s) with the other two time windows and neutral sounds with other sound categories. As can be seen in Figure 5 (bottom row, middle panel), the relative strengths of the responses to anticipation of neutral vs. emotional sounds changed during the anticipation period. The effect of neutral sounds was stronger during the first time window (0.4–0.7 s) and weaker during the last time window (1.5–2 s) than the effect of emotional sounds (between-time-window effect of neutral vs. pleasant sounds *p* = 0.03, effect size r = 0.54; neutral vs. unpleasant *p* = 0.026, r = 0.55). The difference between the first and middle (1–1.5 s) time windows was in between: only the amplitude relation of neutral vs. unpleasant sounds showed a statistically significant difference (*p* = 0.025, r = 0.56). Overall, the emotional sounds had a weaker effect than the neutral sounds during the early time window but had the strongest effect during the late time window. Interestingly, as will be shown below, this relation of the effects during the late time window was the same as the tendency during the hearing period.

### Hearing period 2.5–8 s

During the hearing period, the source strengths were analysed with respect to a 200-ms baseline just preceding the long sounds. This procedure was adopted because source strengths during the anticipation period did not decay to zero before the emotional sounds. The baseline was therefore taken from just before the emotional sound onset to extract responses related to the actual hearing of the emotional sounds but avoiding contamination by response differences during the late anticipation period. Furthermore, as shown in Fig. 1 (bottom), the sound pressures were weaker for unpleasant sounds than for other sound categories during the initial 0.2 s but were very similar for neutral and pleasant sounds after 0.5 s. To avoid effects of these sound pressure differences, we excluded the source strengths within the 2–2.5 s window. Source strengths within the 2.5–8 s hearing period were analysed in one block because visual inspection did not reveal systematic time dependencies, nor were such temporal variations expected.

During the hearing period (2.5–8 s), significant left-hemisphere dominance, [F(1,14) = 6.7, *p*<0.021] was observed for all sounds (Figure 5, period f), with no main effect of category. Comparison of the hemispheric differences of responses to pleasant and unpleasant sounds revealed a tendency towards hemispheric interaction [F(1,14) = 4.02, *p* = 0.067], suggesting that, despite a general left-hemisphere predominance of the signals, left-auditory-cortex responses tended to be stronger to unpleasant than pleasant sounds, whereas the situation was the opposite in the right auditory cortex (Figure 5(f); see also Figure 4, DC-coupled traces).

When the data from both hemispheres were combined (averaging the individual dipole moments from both hemispheres), *category* also had a main effect in one-way ANOVA [*category* (3); F(2,28) = 7.28, *p* = 0.003]. Post-hoc paired t-tests (with Bonferroni correction) showed that pleasant (*p* = 0.048) and unpleasant (*p* = 0.006) sounds both evoked stronger activity than did neutral sounds, with no difference between the pleasant and unpleasant sounds (*p* = 0.9; Figure 5, bottom row, right panel). Thus, the source strengths tended to be larger during emotional than neutral sounds. This tendency is similar to that occurring in the late anticipation period.

## Discussion

We measured cortical auditory-evoked magnetic fields to non-emotional cue tones and to the subsequent emotion-arousing and neutral sounds to assess the effect of emotional content of the sound on auditory-cortex activity, both during anticipation and hearing of the sounds. In our analysis, we focused on both transient and sustained MEG signals, which were clearly visible because of the high temporal resolution of MEG and the applied DC measurements with no high-pass filtering of the signals.

In line with earlier demonstration of auditory-cortex activation before an auditory imperative stimulus [23], the auditory cortices in both hemispheres were activated during the whole silent anticipation period, although the activity became weaker towards the beginning of the emotional and neutral sounds.

During the early time window (0.2–0.35 s), soon after the cue and transient N100m response, the different cue tones had an effect on activation strength in the auditory cortices. During the remaining time windows, the effect faded away and most importantly, did not change towards the end of the anticipation period. However, the significant interaction between *category* and *time window* shown for these later time windows of the anticipation period suggests that the upcoming emotional sound category was responsible for the differences later during the anticipation period. We consider these differences between categories during the anticipation period to reflect implicit associations between the cues and the emotional sounds formed during the training task. Compared with the signals preceding the neutral sounds, the signals preceding the emotional sounds were weaker about 0.5 s after the cue tone, but stronger towards the end of the anticipation period. During the hearing period, the amplitude relations remained the same as during the late anticipation period: both unpleasant and pleasant sounds evoked stronger responses than neutral sounds.

The non-linear dependence of our auditory-cortex signals as a function of stimulus valence agrees with the results of previous studies. Cortical activity of many brain areas may show U-shaped dependence on the emotional valence of applied stimuli [28] so that the effect of neutral stimuli is smaller (or larger) than that of the stimuli with either positive or negative valence. Such U-shaped dependence has also been shown in auditory cortical areas [29], [30]. Accordingly, our results resembled an inverted U-shape during the early time window and an upright U-shape during the late time window of the anticipation period as well as during the hearing period.

When pictures in an fMRI study were first associated with sounds and then presented in isolation, auditory cortices were activated, likely reflecting memory retrieval-related activation of the sensory cortices [31], [32]. Similarly, a part of the observed auditory-cortex activation during the anticipation period may reflect retrieval of the upcoming sound from memory.

During the hearing period, effects of emotion were evident only when the data were pooled from both hemispheres. This weaker-than-expected [10], [33] effect may be due to several factors. First, because of the variety of the emotional sounds used as stimuli (Table 1), the temporal profiles of the sounds, especially around the onsets, were not exactly matched between the categories (Fig. 1). Although previous studies indicated that emotion can be distinguished at the brain level within the first 0.2–0.3 s after sound onset [6], [7], [11], we excluded data for the first 0.5 s from the analysis to avoid contamination of the responses by different early profiles of the sound stimuli. Also, our long time window (2.5–8 s) might have diminished the category effect. Moreover, in contrast to earlier work, our analysis—focused on the auditory cortex generating the N100m response—may have been less sensitive to signals generated in the associative cortex within the superior temporal sulcus or the inferior frontal gyrus areas which have been shown to be more strongly activated by emotional than neutral prosody [4], [5]. Our focus was on brain events during the anticipation period, before the sounds were presented. Since the physical properties of our realistic sounds varied, any brain-response differences between the emotional categories during the sounds (hearing period) could reflect just acoustical differences and are not discussed further here.

Source strengths indicated that the auditory-cortex activity was strongly left–hemisphere lateralized for all sounds, similarly during both anticipating and hearing periods. In the literature, results for hemispheric lateralization of emotion perception, experience, and expression vary according to experimental setups and stimuli (e.g., [34]). One of the popular hypotheses assumes right-hemisphere dominance for the processing of all emotions, regardless of their valence, as is supported, for example, by findings of stronger effects of prosodic emotion (both negative and positive) on the right than the left auditory association areas [29]. According to the valence hypothesis, on the other hand, positive valence dominates processing in the left hemisphere and negative valence in the right hemisphere, especially in the anterior areas (e.g., [34]. In our study, the auditory cortex responded in a left-hemisphere dominant manner to all sound categories but with a tendency towards a stronger effect of unpleasant than pleasant sounds in the left hemisphere and *vice versa* in the right hemisphere. Thus, our data on emotional-sound processing in the auditory cortices neither support the right-hemisphere hypothesis nor the valence hypothesis, suggesting that other brain areas dominate in the lateralization of emotional processing of auditory stimuli.

In conclusion, we have shown that human auditory cortices are bilaterally activated not only during hearing of long emotional and neutral sounds but also while anticipating them. The relative strengths of the auditory-cortex signals during the early and late parts of the anticipation period varied depending on the upcoming sound: towards the end of the anticipation period the activity became stronger when the subject was anticipating emotional rather than neutral sounds. The same trend was observed during the hearing period.

## Acknowledgments

We thank Veikko Jousmäki and Lauri Parkkonen for help in the DC measurements and Pavan Ramkumar for helpful discussions.
